# The assessment of skin sebum and moisture content of infants with atopic dermatitis

**DOI:** 10.3906/sag-1912-56

**Published:** 2020-06-23

**Authors:** Hatice GÜNEŞ, Hülya NAZİK, Mehmet Yaşar ÖZKARS, Nurgül Çimen PARLAK, Ayten YILDIZ, Ömer DUYURAN, Bahaüddin Ahmed YALÇIN

**Affiliations:** 1 Department of Pediatrics, Faculty of Medicine, Kahramanmaraş Sütçü İmam University, Kahramanmaraş Turkey; 2 Department of Dermatology, Faculty of Medicine, Kahramanmaraş Sütçü İmam University, Kahramanmaraş Turkey; 3 Division of Allergy and Immunology, Department of Pediatrics, Faculty of Medicine, Kahramanmaraş Sütçü İmam University, Kahramanmaraş Turkey

**Keywords:** Atopic dermatitis, skin sebum, skin moisture

## Abstract

**Background/aim:**

Atopic dermatitis (AD) is a chronic, inflammatory skin disorder characterized by dry skin, pruritus and eczematous lesions. The aim of this study is to evaluate the skin moisture and sebum content of the infants with AD and its relationship between the disease severity.

**Materials and methods:**

For this cross-sectional and case-control study we included 49 infants with AD aged between 2 and 24 months and 34 healthy infants with the same age and sex as a control group. Skin moisture and sebum content were measured by the bio-impedance method and recorded. Skin prick tests, total IgE levels and food-specific (milk, egg) IgE levels were performed.

**Results:**

There was a significant difference between the groups in terms of mean skin moisture and sebum content percentage (P < 0.001, both). The mean skin moisture and sebum content were correlated with CRP in patients with AD (P < 0.01, r = –1.00, both), but we did not find any correlation between these parameters with the disease severity.

**Conclusion:**

We found that skin moisture and sebum content were low even in unaffected areas in AD. The skin structure of these patients may contribute to the pathogenesis of the disease.

## 1. Introduction 

Atopic dermatitis (AD) is a chronic, relapsing inflammatory skin disorder characterized by scratching eczematous lesions, dry skin, pruritus, and erythematous papules [1, 2]. Recent genetic studies have shown a defective skin barrier in the pathogenesis of AD. This skin barrier in the stratum corneum of the epidermis prevents irritants and allergens from entering the body [1]. The water and the lipid content of skin are both crucial for the skin functions especially barrier function and appearance. The stratum corneum lipids have water-retaining properties that are important for this barrier [3, 4]. There is a balance between the lipids and the water content of the skin. When this balance is disrupted the dry skin occurs which is a common symptom in AD. Additionally skin barrier is also impaired in AD [4].

We aimed to reveal whether AD is a characteristic of moisture and oil balances in the skin that is unique to people with AD, or if there is a tendency to form AD as a result of this imbalance. The aim of this study is to evaluate the skin moisture and sebum content of the infants with AD and compare it to healthy peers, and to examine the relationship between disease severity and other parameters.

## 2. Materials and methods

This cross-sectional and case-control study included 49 infants aged 2–24 months, diagnosed with AD, who were admitted to the pediatric allergy outpatient clinic of Kahramanmaraş Sütçü İmam University. As the control group 34 healthy infants were taken with similar age and sex who admitted to our healthy children outpatient clinic. The children with acute or chronic systemic diseases, infectious diseases, immunodeficiency disorders or any additional dermatologic problems such as seborrheic dermatitis, dermatitis herpetiformis, irritant contact dermatitis, nummular dermatitis scabies, molluscum contagiosum, tinea corporis and capitis, mycosis fungoides, Langerhans cell histiocytosis, pityriasis lichenoides chronica, and psoriasis were excluded from the study.

AD was diagnosed by a child allergy specialist according to the Hanifin-Rajka criteria [5]. AD severity was graded by the severity scoring of atopic dermatitis (SCORAD) index. An objective SCORAD score of <15 was classiﬁed as mild, 15–40 as moderate, and >40 as severe [6]. Total immunoglobulin E levels, food-specific (milk, egg) IgE and skin prick tests were performed to AD group. Skin prick tests were applied to the back of the patients with the same technique by a pediatric allergy specialist. Histamine (10 mg/mL) and physiological saline were used as positive and negative references. Reactions were evaluated 15 min after the application. The positive reaction of 3 mm or larger than the negative control was considered to be the positive test.

A portable pen-shaped LCD Display Digital Skin Moist Oil Analyzer (Reyoung-Beauty, Guangdong, China) was used to measure the sebum and the moisture in the skin. Three different measurements were done from the different body surfaces including the glabella, periumblical area and popliteal fossa. The measurement was made by placing the probe on the bare skin for a few seconds at room temperature. Oil and moisture were obtained in percent and the percentages were recorded. Oil and moisture content between 46% and 43% were considered as moist, 42%–38% as normal, 37%–34% as dry, and 33% and below were considered very dry. After each application, the probes were wiped with soft cloths soaked in alcohol. It was preferred that the patients did not use moisturizing cream, sunscreen or other creams, baby oils or use a coarse bath-glove in the bath/ shower at least 1 day prior to the measurement of skin moisture and sebum. On the day of the measurement, they were asked to wash their face with tap water only once, and not to use cleansing products such as washing gel, and soap. Complete blood counts and C-reactive protein (CRP) levels were performed to study groups and noted.

Informed consent was obtained from all participants’ parents. The study was approved by the local ethical committee.

### 2.1. Statistical analysis

Data management and analysis was performed by using the SPSS program v.14 (SPSS Inc., Chicago, IL, USA) and a two-sided P value ≤0.05 was considered as statistically significant. Categorical variables were determined by the percentage and number of cases, while continuous variables were expressed as the mean ± standard deviation (SD) or median and interquartile range (IQR). A mean was compared by using an independent sample t-test, and in the case of an abnormal distribution Mann–Whitney U test with the median was used. A chi-square test was used for the categorical data. Correlation analyses of abnormally distributed variables were performed by Spearman correlation analysis and Pearson correlation was used in the normal distributed variables.

## 3. Results 

**Figure 1:**
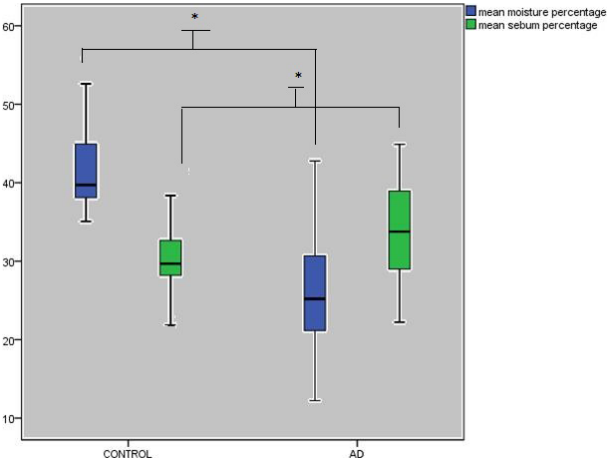
The comparison of mean skin moisture and sebum percentages between the groups; bars represent median (interquartilerange). *P < 0.001,both.

**Figure 2:**
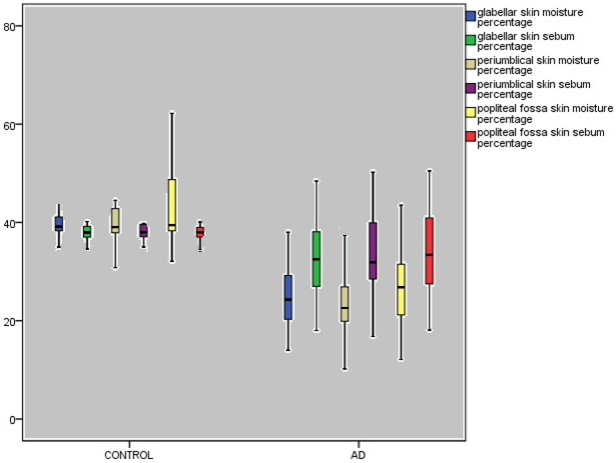
Comparison of skin moisture and sebum percentages of different body parts between the groups; P < 0.001 both for glabellar skin moisture and sebum content percentages, P < 0.001 for periumblical skin moisture percentage, P = 0.001 for periumblical skin sebum percentage, P < 0.001 for popliteal fossa skin moisture percentage, P = 0.078 for popliteal skin sebum percentage, bars represent median (interquartile range).

Table represents demographic and laboratory findings of the study. The median age was similar between the groups while there was a difference in terms of sex. The mean skin sebum (P < 0.001) and moisture percentages (P < 0.001) were significantly lower in AD group compared to those in control group (Figure 1). The comparison of skin moisture and sebum percentages of different body parts between the groups were given in Figure 2. The skin moisture and sebum percentages were low in all body parts in the AD group and this is statistically significant except popliteal fossa sebum percentage (P = 0.078). The white blood cell (WBC) and eosinophil counts and CRP were significantly higher in AD group. There were 5 patients whose egg specific IgE was positive, 5 have milk specific IgE positive and 6 have both milk and egg positive, while 32 of them have negative results. When the patients were grouped according to their sex, we found that the mean skin moisture and sebum percentages were not different in both of the groups (P = 0.063, P = 0.359).While the skin prick test of 5 patients were positive and 37 were negative, the others have not got test results because their families did not give consent for testing. When the patients with AD were divided into groups according to skin prick tests as positive and negative, we found that the mean skin moisture and sebum percentages were not different between the groups (P = 0.881, P = 0.960, respectively).

**Table:** Baseline characteristics of study patients.

	AD group(n = 49)	Control group (n = 34)	P
Age, months a	7 (4–11)	6.5 (3–15)	0.179
Sex, male/female (n)	34/15	16/18	0.041*
Glabellar skin moisture percentage b	26.31 ± 10.58	41.52 ± 7.24	0 < 001
Glabellar skin sebum percentage b	32.71 ± 7.62	37.93 ±4.79	0 < 001
Periumblical skin moisture percentage a	22.60 (19.70–27.35)	39.05 (37.90–42.95)	0 < 001
Periumblical skin sebum percentage b	33.87 ± 7.50	38.33 ± 4.25	0.001
Popliteal fossa skin moisture percentage a	26.80 (21.10–31.60)	39.45 (38.27–48.75)	0 < 001
Popliteal fossa skin sebum percentage b	34.76 ± 8.36	37.39 ± 5.00	0.078
Average skin moisture percentage b	26.37 ± 7.07	42.35 ± 6.20	0 < 001
Average skin sebum percentage b	33.78 ± 5.65	37.88 ± 2.48	0 < 001
WBC, 10³ mm³ b	10.52 ± 3.16	9.10 ± 2.82	0.044
Neutrophil, mm³ a	2.06 (1.51–3.23)	1.82 (1.29–2.94)	0.878
Lymphocyte, mm³ b	5.90 ± 2.42	5.25 ± 2.08	0.217
Eosinophils, mm³ a	0.48 (0.31–0.80)	0.26 (0.15–0.43)	0.001
Monocyte, mm³ b	1.04 ± 1.03	0.85 ± 0.43	0.340
Platelet count, 10³mm³ b	430.31 ± 111.90	405.68 ± 81.71	0.289
MPV, fl b	9.72 ± 0.78	9.88 ± 0.79	0.372
CRP, mg/dl a	4.51 (3.23–4.50)	3.20 (3.20–3.20)	0<001
SCORAD score b	39.28 ± 18.26		
Total IgE a	18.90 (18.80–30.70)		

a Median (interquartile range), b Mean ± standard deviation, * Chi-square. CRP: C-reactive protein, MPV: Mean platelet volume, WBC: White blood cell count.

While the mean skin moisture and sebum percentages were correlated with CRP in patients with AD (P < 0.001, r = –1.00, both), we found no significant correlation between these parameters and SCORAD (P = 0.115, r = 0.241, P = 0.732, r = –0.053, respectively). SCORAD was correlated only with periumblical skin moisture (P = 0.026, r = 0.337), CRP (P < 0.001, r = 1.00) and, IgE (P = 0.016, r = 0.371).

## 4. Discussion

In this study, we compared the skin moisture and sebum percentages with healthy peers in order to understand the skin nature of infants with AD. To better illustrate this, we conducted this research by excluding body areas with frequent involvement in AD. We revealed the mean skin sebum and moisture percentages were significantly different from each other. Mean moisture percentage was low in AD group. This situation has been found with different results in different studies. In the studies of Tupker et al., Werner et al. and Sator et al. on patients with AD, they found that the water content of the skin was low [4, 7, 8]. Also, there are contrary studies about the high water content of the skin of AD patients in the unaffected body parts, compared to the healthy one [9,10]. Skin sebum also plays an important role in maintaining moisture and contributing to skin integrity. The sphingolipids and other neutral lipids in the stratum corneum provide the integrity of the skin barrier due to their water retention properties, thereby protecting the body from external irritants [4]. Therefore, topical moisturizers are the basis of AD treatment because they increase the water retention of the skin and prevent water loss [11]. Sator et al. showed that patients with AD had lower skin fat [4]. Similarly we found low skin sebum percentages in the AD group.The percentage of skin sebum was low in all body areas except popliteal fossa. Popliteal region is the region that is not expected to be affected in this age group, and no significant difference can be attributed to this. However, even if there is no significant difference, it is extremely important to be lower than the control group. Because, with the increase in age, we think that sebum level can be used in the prediction before clinical findings, since clinical findings are expected in this region.

Mean skin moisture and sebum percentages were negatively correlated with CRP in AD group. This may be related to the current systemic inflammatory state in AD, as shown in previous studies [12–14]. On the other hand, mean skin moisture and sebum did not correlate with SCORAD, which may be due to measurements taken from areas where no involvement was observed. But SCORAD was correlated only periumblical skin moisture, which may be due to the fact that this part of body area is more protected against external influences such as cold, dry air compared to other measured regions. Similar to our study, Gupta et al. showed transepidermal water loss is correlated with AD severity in normal appearing nonlesional skin areas [1]. This finding supports an intrinsic defect causes the pathogenesis of AD. SCORAD is also correlated with IgE and CRP in our study. This finding supported by the study of Vekaria et al. who demonstrated that CRP levels were high in AD group according to control group and also they showed a correlation between CRP and SCORAD [15]. Although skin prick tests of patients with food allergy are known to be positive and specific IgE are known to be high, food allergen-specific T cells have been cloned from the skin of patients with AD [16]. This shows that food allergens also contribute to the immune response and supports our findings. However, although it is difficult to say which food or allergen type has more skin involvement in AD with the available data, when the patients were grouped according to skin prick tests as positive and negative, there was no difference between skin moisture and sebum percentages between the 2 groups. Although there was a sex difference between the groups, we found that skin moisture and sebum percentages were similar when we classified patients according to sex.

The limited number of cases and the fact that our measurements were not made from additionally affected regions in AD cases can be considered as limitations of our study.

In conclusion, we found that skin moisture and sebum were low even in unaffected areas in AD. We think that the skin structure of these patients may contribute to the pathogenesis of the disease.

## Financial disclosure

The authors declared that this study has received no financial support.

## Conflict of interest

No conflict of interest was declared by the authors.
